# The association of solitary versus group-based arts participation with mental health: evidence from the UK Household Longitudinal Study

**DOI:** 10.1177/14034948251370234

**Published:** 2025-10-03

**Authors:** Gökhan Kaya, Christopher Mathieu

**Affiliations:** Department of Sociology, Lund University, Lund, Sweden

**Keywords:** Adult, art, culture, longitudinal studies, mental health, health, social participation, survey and questionnaires, United Kingdom

## Abstract

**Aims:**

Research on the role of art in alleviating mental health problems has increased dramatically. However, it remains unclear whether mental health benefits of the arts are the same across different social contexts of arts participation. The aim of the study is to investigate associations between two modes of arts participation, solitary and group-based, and psychological wellbeing (12-item general health questionnaire (GHQ-12).

**Methods:**

Data from waves 2 and 5 of the United Kingdom Household Longitudinal Study, involving 23,706 respondents, are used. Fixed-effects ordinary least squares is applied to examine the associations by following the same individuals over time.

**Results:**

Our results show a positive association between mental health – using the GHQ-12 psychological wellbeing scale – and frequent participation in group-based arts activities (*b*=0.45, 95% confidence interval (CI) 0.19 to 0.71, *P*<0.001). In contrast, solitary arts activities are not significantly associated with psychological wellbeing, even for those who participate frequently (*b*=0.20, 95% CI −0.04 to 0.44, *P*>0.05). The results remain similar when controlling for key social determinants of mental health such as unemployment and social support, and when running robustness checks using two other outcomes: life satisfaction and mental health functioning (12-item short form health survey mental health component).

**Conclusions:**

**Arts participation and social context work in tandem and group-based participation is more effectively associated with lower levels of mental health problems. The results show more beneficial outcomes for group-based as opposed to solitary arts participation, proving this distinction is important for further research and providing a key insight for arts-based social prescribing.**

## Background

Art is increasingly recognised as a means of alleviating mental health problems including anxiety and depression [1], as a healing tool for cancer patients [2], and for helping trauma recovery for specific groups like refugees [3]. In the United Kingdom, art-related social prescribing is increasing, aimed at improving mental health [4, 5]. Although more large-scale surveys now examine art`s role in mental health among the general population and subgroups like the elderly [6–9], findings regarding associations between mental health and arts participation remain inconclusive. Some studies report stronger associations between receptive arts activities and mental health than arts participation [6, 9, 10] while others show mixed results, such as increased arts participation being linked to poorer health, including more bodily pain and lower vitality [7], and no clear relationship with depressed mood [8].

These inconclusive findings may stem from viewing arts participation as a singular activity (see DiMaggio [11]), without distinguishing between solitary and group-based arts engagement. This insufficient distinction between social contexts muddles our understanding of the role of arts participation on mental health. Participation in arts activities unfolds across different social contexts, shaped by the degree of social focus they involve and the opportunities they create for meeting like-minded people, forming social bonds, and developing a sense of togetherness [12–14]. Group-based practices such as dance performances, choral singing, and theatre require regular rehearsals and collective performances, naturally fostering social bonds. Other arts activities, like crafting, painting, or composing music, are typically solitary, with over half of participants in a UK study reporting engagement mainly alone [15]. Although solitude fulfils human needs and does not imply isolation, solitary arts practice offers fewer meeting opportunities to build social bonds and a sense of togetherness than group-based activities. This difference also matters when facing arts-related stressors like performance anxiety, fear of failure, and need for recognition [16]. While group-based arts engagement involves similar stressors, it provides more opportunities for mutual coping. While solitary engagement offers autonomy and recognition, it places more individual responsibility under creative pressure. Taken together, we expect that mental health benefits of arts participation depend on whether it is group-based or solitary. Evidence for mental health benefits of group-based arts activities often comes from small sample qualitative studies focusing on specific arts activities [17, 18]. Using the UK Household Longitudinal Study (UKHLS), the following question guides our research: To what extent is arts participation associated with mental health, and do these associations differ between group-based and solitary participation?

### Aims

The aim of the study is to test whether group-based and solitary arts participation have positive associations with mental health using the 12-item general health questionnaire (GHQ-12), a widely used mental health indicator, with robustness checks using life satisfaction and mental health functioning. To our knowledge, this is the first study to examine the mental health benefits of group versus solitary arts engagement using longitudinal data at the population level, providing systematic evidence to inform arts-based social prescriptions.

## Methods

### Study design and participants

**Figure 1.**
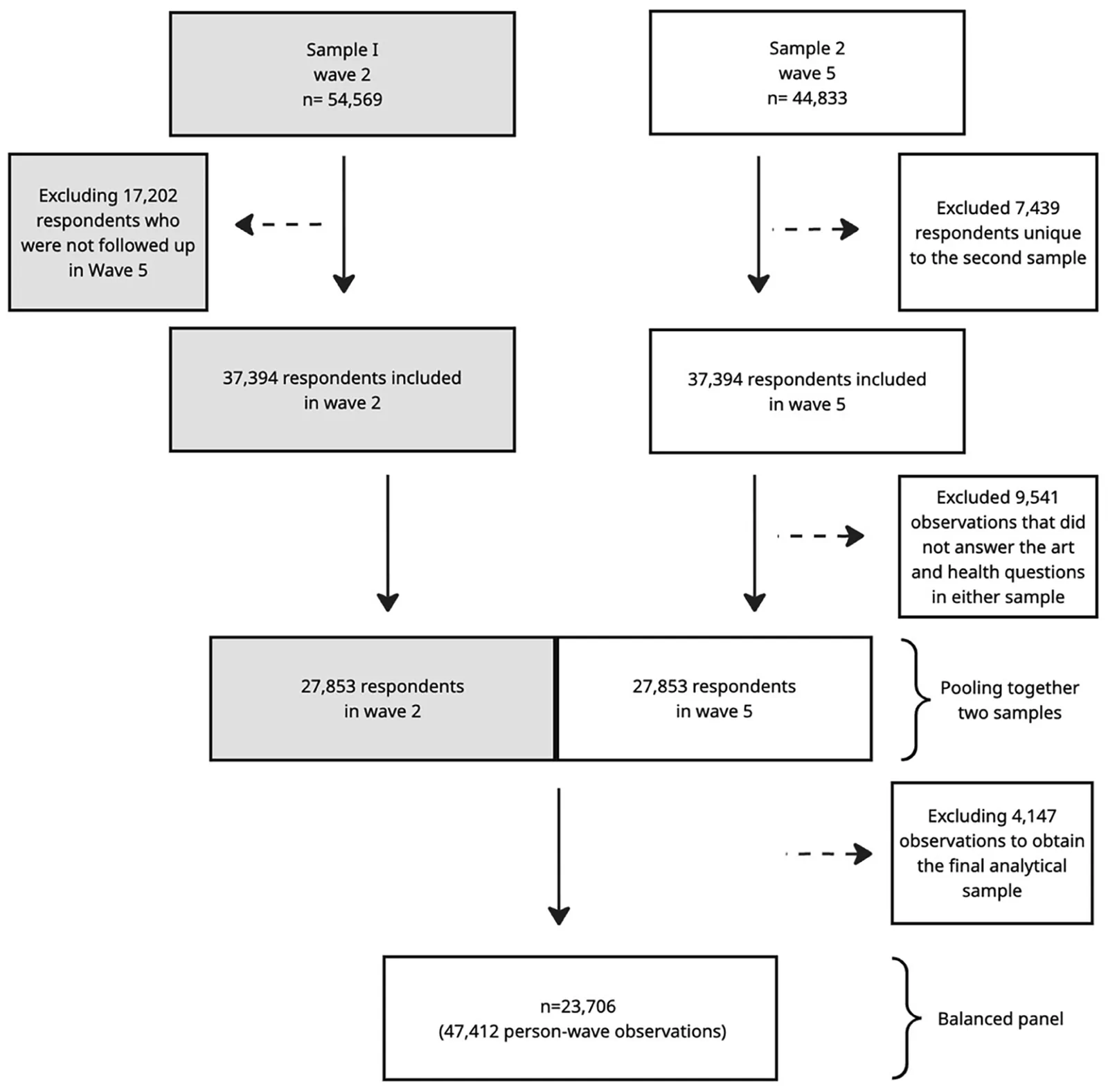
Sample characteristics. Sample characteristics are illustrated primarily for the focal associations between psychological wellbeing (general health questionnaire (GHQ-rev)) and group-based/solitary arts participation, including control variables. Sample size varies in robustness checks using listwise deletion for life satisfaction and 12-item short form health survey (SF-12) mental health functioning (mental component summary; MCS). See Table I.

The UKHLS provides a nationally representative probability sample [19]. The UKHLS covers a wide range of topics such as education, family, health, income, and work. Respondents aged 16+ years in the household complete the questionnaire. We use a panel design across two waves including the same individuals over approximately 3 years: Wave 2 (*n*=54,569, January 2010 to March 2012) and wave 5 (*n*=44,833, January 2013 to June 2015). These waves were chosen because they include arts participation and mental health variables. Figure 1 shows 69% were followed from wave 2 to wave 5 (*n*=37,394). To obtain a balanced panel, we excluded missing values in arts or mental health variables in both waves (9541) and in control variables within the analytical sample (4147), resulting in a final sample of 23,706 (Figure 1).

**Table I.** Descriptive statistics for the variables used in the analysis (wave 2 and wave 5).

	Wave 2				Wave 5			
	*n*	Freq.	Mean/%	SD	*n*	Freq.	Mean/%	SD
Group-based arts participation (%)	23,706				23,706			
None		19,085	80.5			19,868	83.8	
Once/twice a year		732	3.1			647	2.7	
At least once a month		843	3.6			626	2.6	
At least once a week		3046	12.8			2565	10.8	
Solitary arts participation (%)	23,706				23,706			
None		15,856	66.9			16,120	68.0	
Once/twice a year		1011	4.3			973	4.1	
At least once a month		1404	5.9			1385	5.8	
At least once a week		5435	22.9			5228	22.1	
Attending arts events (%)	23,706				23,706			
None		4771	20.1			5086	21.5	
At least once/twice a year		24.4	0.24			25.5	0.25	
Several times a year		8641	36.5			8413	35.5	
At least once a month		4514	19.0			4172	17.6	
GHQ-12 reversed (0–36)	23,706		25.07	5.21	23,706		24.97	5.38
Life satisfaction (1–7)	23,546		5.31	1.41	23,546		5.10	1.48
SFL-12 MCS: Mental health functioning (0–100)	22,085		50.41	9.13	22,085		49.67	9.54
Age (16–103)	23,706		47.45	16.79	23,706		50.47	16.78
Marital status (%)	23,706				23,706			
Single		6256	26.4			5577	23.5	
Married		13,344	56.3			13,708	57.8	
Divorced		2756	11.6			2857	12.1	
Widowed		1350	5.7			1564	6.6	
Child under 16 years (0–1)	23,706		0.47	0.34	23,706		0.25	0.43
Employment status (%)	23,706				23,706			
Employed		14,196	59.9			14,175	59.8	
Unemployed		1006	4.2			778	3.3	
Inactive		8504	35.9			8753	36.9	
Level of education (%)	23,706				23,706			
University degree		5962	25.1			6423	27.1	
Other higher degree		3025	12.8			3157	13.3	
A-level etc.		4848	20.5			4774	20.1	
GCSE etc.		5041	21.3			4720	19.9	
Other qualification		2272	9.6			2227	9.4	
No qualification		2558	10.8			2405	10.1	
Log income (0–14.5)	23,706		10.49	0.76	23,706		9.80	0.73
Family support (1–4)	23,706		3.15	0.75	23,706		3.14	0.76
Friend support (1–4)	23,706		3.15	0.72	23,706		3.12	0.74
Long-standing health condition (0–1)	23,706		0.34	0.47	23,706		0.34	0.47
Sport activity (0–10)	23,706		3.58	2.86	23,706		3.32	2.87

Frequencies and percentages are reported for categorical variables while mean and SDs (standard deviations) are reported for continuous variables.

Min–max values are in parentheses for each continuous variable.

The numbers are unweighted.

### Measures

#### Outcome measures

We use three indicators of mental health: psychological wellbeing, life satisfaction, and mental health functioning. Psychological wellbeing is measured using the GHQ-12, an instrument used in psychiatry for screening multidimensional mental health [20]. The 12 items include lists of symptoms such as depression, anxiety, and sleep difficulties. Each symptom is reported for occurrence during ‘the past few weeks’ using a four-point scale (less than usual, no more than usual, rather more than usual, much more than usual). We reverse the GHQ-12 scale, indicating higher scores for better psychological wellbeing, showing strong internal consistency (0.90 at waves 2 and 5). Life satisfaction is measured with a single item, evaluating overall satisfaction with one’s current situation on a seven-point Likert scale from completely dissatisfied (1) to completely satisfied (7). The survey includes eight indicators derived from the 12-item short form health survey (SF-12) that measure quality of life, namely: physical functioning, role limitations due to physical health problems, bodily pain, general health, vitality, social functioning, role limitations due to emotional problems, and mental health. In the UKHLS, the SF-12 mental component summary (MCS) score is calculated by giving greater weight to the latter six. It ranges from 0 to 100, where higher values reflect better mental health functioning. It exhibits high internal consistency (α=0.90).

#### Explanatory variables

The dataset includes two sets of arts-related leisure activities, comprising 13 dummy variables (mentioned or not mentioned) (see Supplemental Table I). The follow-up question included five frequency categories for arts participation (separating once and twice a year), which we recoded into four: (i) none, (ii) once or twice a year, (iii) at least once a month, and (iv) once per week or more.

We classified the dummy variables as either group-based or solitary. Group-based arts activities refer to activities predominantly carried out in collaborative settings: dance including ballet; learning or practising circus skills; rehearsing or performing in a play, drama, opera, operetta, or musical theatre; singing to an audience or rehearsing for a performance (but excluding karaoke); or taking part in a carnival or street arts event as a musician, dancer, or costume-maker. Solitary arts activities refer to arts activities predominantly done alone (see Tymoszuk et al. [15]): painting, drawing, printmaking, or sculpture; textile, wood, and other crafts, including knitting and wood-turning; making furniture, pottery, or jewellery; using a computer to create original artworks or animation; or writing stories, plays, and poetry. Based on this classification, we constructed two new variables: group-based arts engagement and solitary arts engagement. Group-based arts engagement was coded if respondents participated in at least one group-based activity. Respondents who reported engagement only in activities other than arts activities in a group setting were recoded as ‘none’. We applied the same steps to create solitary arts engagement, using the four-category frequency scale. Spearman’s rank order correlations indicated a weak positive association between the two variables (ρ=0.215 in wave 2; ρ=0.214 in wave 5), suggesting that while related, they capture distinct aspects of arts participation.

#### Control variables

Attending arts events shows frequencies (none, at least once or twice a year, several times a year, at least once a month) for attending at least one of the following arts events: film screenings, art exhibitions, photography or sculpture displays; book-related events; street art displays; theatrical performances such as plays, pantomimes, and musicals; operas; classical music concerts; rock, pop, or jazz performances; ballet or contemporary dance; or African, South Asian, or Chinese dance events. Time-fixed effects refer to a dummy variable for wave 5 (reference category: wave 2). The sociodemographic controls are age, aged squared, educational level (no qualification, other qualification, General Certificate of Secondary Education (GCSE), A-level, other higher degree, university degree), log-transformed household income, marital status (single, married, divorced, widowed), child under 16 years (yes, no). Long-standing health conditions refer to self-reported physical or mental impairment, illness, or disability over 12 months (yes, no). Family support and friend support are two summated indices derived from three similar questions: can talk about my worries with family/friends; can rely on family/friends; family/friends understand the way I feel. The indices range from 1 (not at all) to 4 (a lot). Sports activity ranges from 0 (no sport at all) to 10 (very active through sport).

### Statistical analysis

We use fixed-effect ordinary least squares (OLS) to estimate associations between group-based and solitary arts participation with psychological wellbeing. Using OLS models with cross-sectional data is limited in controlling for the confounding effects of unobserved variables, introducing bias into estimates. Fixed-effects models reduce omitted variable bias by adjusting for time-invariant characteristics. Fixed-effect models are primarily used to estimate the effect of time-varying characteristics – changes in certain variables over time within the same individual that are linked to changes in the outcome [21]. The Hausman test supported the selection of a fixed effects model instead of a random effects model. Due to significant heteroscedasticity detected by the modified Wald test, sandwich estimators were used to adjust standard errors. Our analysis applies longitudinal weights to correct for standard errors due to non-response and the sampling design.

The models control for the following variables: attending arts events; time-fixed effects; sociodemographic factors including age, age squared, marital status, presence of children under 16 years, employment status, household income (logged), and education level; and social determinants of mental health, including long-standing health conditions, family support, friends’ support, and health behaviours such as sports activity. Due to the non-linear relationship, both age and age squared were included as predictors (see full models in Supplemental Table IV). Logarithmic transformation of income reduces outlier impacts. Time-fixed effects control for time-specific changes in the outcome.

We conducted two robustness checks. The first explores mental health outcomes using life satisfaction and mental health functioning. The second checks the results using alternative measures of group-based and solitary arts participation. This measure is consistent with Tymoszuk et al. [15], showing that over half of participants in the UK engage in activities like crafting, painting, and writing music mostly alone. The same study shows that while some activities, such as playing a musical instrument, are often done in solitude rather than in groups, they can also be done in both solitary and group settings. This suggests that such activities do not align perfectly with the binary classification of solitary versus group based. Therefore, we examined four alternative measures, including and excluding such variables, as well as creating mixed activities, and found similar results (see Supplemental Tables II and III).

## Results

Table II shows the profiles of individuals by arts participation frequency. It shows that individuals who participate most frequently in group-based arts are more socioeconomically advantaged. Around 25% and 27% of individuals in each wave were university educated (see Table I) and participated more in arts weekly, whether group-based or solitary (see Table II). The data reveal that prevalence of unemployment is lower among group-based arts participants. In the first sample, the unemployment rate is 4.20% (see Table I), dropping to 3.4% for frequent group-based arts and 4.1% for frequent solitary arts participants (a similar trend in the second sample).

**Table II.** Profile of respondents participating in group-based or solitary arts activities at varying frequencies in wave 2 and wave 5.

Variables	Wave 2								Wave 5							
	Arts participation (group-based)				Arts participation (solitary)				Arts participation (group-based)				Arts participation (solitary)			
	None	Once/ twice a year	At least once a month	Once per week or more	None	Once/ twice a year	At least once a month	Once per week or more	None	Once/ twice a year	At least once a month	Once per week or more	None	Once/ twice a year	At least once a month	Once per week or more
Age (16–103)	47.81 (16.73)	44.42 (16.62)	43.33 (15.99)	47.10 (17.17)	47.93 (16.47)	42.62 (16.24)	42.91 (16.48)	48.13 (17.55)	50.62 (16.88)	47.15 (15.66)	45.58 (15.82)	51.33 (16.28)	50.75 (16.74)	45.37 (16.03)	45.18 (15.80)	51.99 (16.86)
Marital status (%)																
Single	25.69	31.83	32.15	27.91	25.10	33.43	32.98	27.14	23.42	25.50	30.19	22.22	23.14	29.70	29.31	22.04
Married	56.98	53.83	53.26	53.41	57.34	53.02	53.35	54.59	57.99	57.50	52.88	57.86	58.04	56.22	55.81	58.00
Divorced	11.52	11.07	11.15	12.57	11.91	10.09	10.04	11.48	11.87	13.29	13.10	12.90	12.21	9.97	10.83	12.28
Widowed	5.82	3.28	3.44	6.11	5.64	3.46	3.63	6.79	6.72	3.71	3.83	7.02	6.61	4.11	4.04	7.69
Child under 16 years (0–1)	0.35 (0.48)	0.35 (0.48)	0.36 (0.48)	0.29 (0.45)	0.34 (0.47)	0.41 (0.49)	0.40 (0.49)	0.30 (0.46)	0.25 (0.44)	0.29 (0.45)	0.25 (0.43)	0.19 (0.39)	0.25 (0.43)	0.35 (0.48)	0.31 (0.47)	0.22 (0.41)
Employment status (%)																
Employed	59.20	65.44	69.51	60.18	61.98	61.62	61.40	53.06	59.10	71.10	70.61	59.69	60.90	66.29	65.78	53.60
Unemployed	4.39	5.05	3.44	3.38	4.17	5.24	5.06	4.07	3.37	3.09	3.04	2.73	3.33	3.29	2.96	3.21
Inactive	36.42	29.51	27.05	36.44	33.85	33.14	33.55	42.87	37.53	25.81	26.36	37.58	35.77	30.42	31.26	43.19
Level of education (%)																
University degree	23.37	24.32	33.45	34.21	22.94	21.17	29.49	31.22	25.12	28.59	40.10	38.87	24.00	31.45	35.31	33.65
Other higher degree	12.38	15.03	14.12	14.22	11.68	15.13	13.68	15.23	12.96	16.85	13.74	15.09	12.21	14.18	14.73	16.18
A-level etc.	20.39	19.67	21.95	20.62	20.28	22.55	24.07	19.61	20.12	22.57	20.93	19.45	20.16	22.10	23.32	18.88
GCSE etc.	21.77	23.50	18.15	18.42	22.23	22.55	21.15	18.25	20.70	18.24	13.58	15.79	21.46	19.53	15.52	16.37
Other qualification	10.21	9.56	7.12	6.37	10.49	9.79	6.20	7.76	10.01	8.50	6.07	5.69	10.36	7.91	6.06	7.57
No qualification	11.88	7.92	5.22	6.17	12.38	8.80	5.41	7.91	11.10	5.26	5.59	5.11	11.81	4.83	5.05	7.35
Log income (0–14.5)	10.47 (0.75)	10.53 (0.77)	10.61 (0.76)	10.55 (0.77)	10.49 (0.76)	10.49 (0.72)	10.54 (0.69)	10.47 (0.78)	9.78 (0.72)	9.86 (0.67)	9.95 (0.68)	9.84 (0.80)	9.80 (0.72)	9.84 (0.67)	9.86 (0.72)	9.76 (0.76)
Family support (1–4)	3.14 (0.75)	3.12 (0.73)	3.14 (0.74)	3.21 (0.72)	3.14 (0.75)	3.13 (0.75)	3.11 (0.74)	3.18 (0.74)	3.13 (0.77)	3.19 (0.71)	3.15 (0.75)	3.17 (0.75)	3.13 (0.77)	3.14 (0.75)	3.13 (0.75)	3.16 (0.76)
Friend support (1–4)	3.12 (0.73)	3.22 (0.68)	3.23 (0.64)	3.28 (0.66)	3.13 (0.73)	3.12 (0.71)	3.19 (0.68)	3.22 (0.69)	3.10 (0.75)	3.14 (0.74)	3.22 (0.70)	3.25 (0.69)	3.09 (0.75)	3.11 (0.74)	3.17 (0.73)	3.20 (0.71)
Long-standing health conditions (0–1)	0.35	0.29	0.30	0.33	0.33	0.33	0.32	0.38	0.35	0.28	0.27	0.32	0.33	0.31	0.31	0.38
	(0.48)	(0.45)	(0.46)	(0.47)	(0.47)	(0.47)	(0.47)	(0.48)	(0.48)	(0.45)	(0.45)	(0.47)	(0.47)	(0.46)	(0.46)	(0.49)
Sports activity (0–10)	3.39 (2.87)	4.13 (2.77)	4.29 (2.70)	4.45 (2.70)	3.50 (2.91)	3.66 (2.79)	3.91 (2.73)	3.72 (2.76)	3.16 (2.86)	3.91 (2.75)	4.12 (2.68)	4.17 (2.77)	3.24 (2.91)	3.50 (2.75)	3.60 (2.73)	3.43 (2.79)

Percentages are reported for categorical variables, while means and standard deviations (SDs) are reported for continuous variables.

Min–max values are in parentheses for each continuous variable.

The numbers are unweighted.

As the same individuals participate in both samples, they age and tend to live in a partnership (see Table II). The average age rises from 47.45 to 50.47 years across samples (see Table I), with slight differences by arts participation frequency. In wave 2, singles are more likely to engage frequently in arts than those who do not participate at all, compared with married individuals or those with children under 16 years. Notably, individuals with long-standing health conditions are less likely to participate in group-based arts activities but are overrepresented in solitary arts, suggesting constraints in activities like dance and singing (see Table II).

Arts participants, whether solitary or group-based, report higher support from friends and family than non-participants and the sample average. For example, the average support from friends and family is 3.15 in wave 2 (see Table I). This number is higher for individuals who participate in arts at least once a week in both settings. A similar pattern is observed in wave 5. Finally, individuals involved in group-based arts participation engage in sports more often than the average respondents. In wave 2 (as in wave 5), those who engage in group-based arts participation once a week or more have a sports activity score of 4.45, compared with 3.72 for those who practice arts in solitude once a week or more, and 3.58 for all respondents (see Tables I and II). Thus, while solitary arts participants are less physically active than group-based participants, they still engage in sports more than the average respondent.

Having described the participants’ profiles, we proceed to the research question. Table III and Table IV present fixed effect OLS models examining the associations between arts participation and mental health. We focus on the coefficients for frequent arts participations, comparing them with those who do not participate in arts at all. The main finding in Table III is that group-based arts participation is significantly associated with higher psychological wellbeing. Model 1 presents the association, with an estimated coefficient of 0.45 (95% confidence interval (CI) –0.19 to 0.71, *P*<0.001). In model 2, with controls, the association decreases to 0.37 (95% CI 0.11 to 0.62, *P*<0.001). Results remain similar when controls are added sequentially: socioeconomic background, health behaviours, and support (see Supplemental Table IV).

**Table III.** Fixed effect OLS predicting the association between group-based arts participation and psychological distress (GHQ-12 reversed), life satisfaction and SFL-12 MCS: mental health functioning with controls (coefficients, confidence intervals are in parenthesis).

	GQH-rev		Life satisfaction		SFL-12 MCS: mental health functioning	
	M1	M2	M3	M4	M5	M6
Arts participation (group-based) (ref.cat=none)						
Once/twice a year	–0.23 [–0.65, 0.18]	–0.28 [–0.69, 0.12]	–0.05 [–0.19, 0.08]	–0.08 [–0.21, 0.06]	–0.25 [–0.93, 0.43]	–0.34 [–1.00, 0.32]
At least once a month	0.34 [–0.08, 0.77]	0.30 [–0.12, 0.72]	0.09 [–0.03, 0.20]	0.07 [–0.05, 0.18]	0.27 [–0.50, 1.04]	0.22 [–0.54, 0.97]
At least once a week	0.45*** [0.19, 0.71]	0.37*** [0.11, 0.62]	0.12*** [0.04, 0.20]	0.10** [0.02, 0.17]	0.62*** [0.16, 1.08]	0.49** [0.04, 0.95]
Wave 5 (ref. cat=wave 2)	–0.10** [–0.19,–0.01]	–1.11** [–2.14, –0.08]	–0.21*** [–0.23, 0.18]	–0.55*** [–0.86, –0.23]	–0.83*** [–0.99, –0.67]	–0.84 [–2.73, 1.05]
Attending arts events (ref.cat=none)						
At least once/twice a year		0.08 [–0.14, 0.30]		0.12*** [0.05, 0.18]		0.37* [–0.03, 0.78]
Several times a year		0.17 [–0.06, 0.41]		0.17*** [0.10, 0.25]		0.26 [–0.18, 0.70]
At least once a month		0.31**		0.17***		0.48*
Employment status (ref.cat= employed)		[0.02, 0.60]		[0.09, 0.26]		[–0.03, 1.00]
Unemployed		–1.48*** [–1.98, –0.99]		–0.27*** [–0.41, –0.12]		–1.02** [–1.89, –0.15]
Inactive		–0.34** [–0.62, –0.05]		0.00 [–0.08, 0.08]		–0.22 [–0.73, 0.29]
Long-standing health condition (ref.cat=none)						
Yes		–0.79*** [–1.00, –0.59]		–0.15*** [–0.20, –0.09]		–0.98*** [–1.33, –0.62]
Family support		0.61*** [0.47, 0.74]		0.10*** [0.07, 0.14]		1.06*** [0.81, 1.30]
Friend support		0.34*** [0.20, 0.48]		0.11*** [0.07, 0.15]		0.49*** [0.24, 0.74]
Sport activities		0.06*** [0.02, 0.10]		0.01** [0.00, 0.03]		0.09*** [0.03, 0.16]
Number of observations	47,412	47,412	47,092	47,092	44,170	44,170
Number of groups	23,706	23,706	23,546	23,546	22,085	22,085
R-squared for within model	0.00	0.03	0.02	0.03	0.01	0.03
Intraclass correlation (rho)	0.58	0.76	0.51	0.78	0.60	0.59

The results are weighted to correct non-response, and unequal sample selection.

The longitudinal weights are provided by UKHLS.

Models M2, M4, and M6 also control for the following variables: age, age squared, education dummies, logged household income, and marital status dummies.

* *P*<0.05; **P<0.01; ***P<0.001.

**Table IV.** Fixed-effect OLS predicting the association between solitary arts participation and psychological wellbeing (GHQ-12 (reversed)), life satisfaction and SFL-12 MCS: mental health functioning with controls (coefficients, confidence intervals in parentheses).

	GHQ-rev		Life satisfaction		SFL-12 MCS: mental health functioning	
	M1	M2	M3	M4	M5	M6
Arts participation (solitary) (ref.cat=none)						
Once/twice a year	–0.10 [–0.46, 0.27]	–0.10 [–0.46, 0.26]	–0.08 [–0.19, 0.03]	–0.08 [–0.19, 0.03]	–0.05 [–0.67, 0.57]	–0.06 [–0.67, 0.55]
At least once a month	–0.23 [–0.53, 0.08]	–0.20 [–0.50, 0.11]	–0.05 [–0.14, 0.03]	–0.06 [–0.14, 0.03]	–0.42 [–0.94, 0.10]	–0.38 [–0.89, 0.13]
At least once a week	0.20 [–0.04, 0.44]	0.18 [–0.05, 0.42]	0.03 [–0.03, 0.09]	0.02 [–0.04, 0.09]	0.21 [–0.17, 0.59]	0.16 [–0.22, 0.53]
Wave 5 (ref. cat=wave 2)	–0.11** [–0.20, –0.02]	–1.14** [–2.17, –0.10]	–0.21*** [–0.24, –0.18]	–0.55*** [–0.86, –0.24]	–0.84*** [–1.00, –0.68]	–0.87 [–2.76, 1.03]
Attending arts events (ref.cat=none)						
At least once/twice a year		0.08 [–0.14, 0.30]		0.12^***^ [0.05, 0.19]		0.38^*^ [–0.03, 0.78]
Several times a year		0.18 [–0.06, 0.42]		0.18^***^ [0.10, 0.25]		0.27 [–0.17, 0.71]
At least once a month		0.32** [0.03, 0.61]		0.18*** [0.09, 0.26]		0.50* [–0.02, 1.01]
Employment status (ref.cat=employed)						
Unemployed		–1.49*** [–1.99, –0.99]		–0.27*** [–0.41, –0.13]		–1.02** [–1.90, –0.15]
Inactive		–0.34** [–0.62, –0.05]		0.00 [–0.08, 0.08]		–0.22 [–0.73, 0.28]
Long-standing health condition		–0.80*** [–1.00, –0.59]		–0.15*** [–0.20, –0.09]		–0.98*** [–1.33, –0.63]
Family support		0.60*** [0.47, 0.74]		0.10*** [0.07, 0.14]		1.05*** [0.81, 1.30]
Friend support		0.35*** [0.21, 0.49]		0.11*** [0.07, 0.15]		0.49*** [0.24, 0.74]
Sports activities		0.06*** [0.02, 0.10]		0.01** [0.00, 0.03]		0.10*** [0.03, 0.16]
Number of observations	47,412	47,412	47,092	47,092	44,170	44,170
Number of groups	23,706	23,706	23,546	23,546	22,085	22,085
R-squared for within model	0.02	0.02	0.02	0.03	0.01	0.03
Intraclass correlation (rho)	0.58	0.77	0.53	0.78	0.61	0.60

The results are weighted to correct non-response, and unequal sample selection.

The longitudinal weights are provided by UKHLS.

Models M2, M4, and M6 also control for the following variables: age, age squared, education dummies, logged household income, and marital status dummies.

* *P*<0.05; **P<0.01; ***P<0.001.

To ease the interpretation of the coefficient and effect size in Table III, we compare the numbers with the stronger predictor of mental health. Ordering the absolute standardised coefficients in decreasing order, unemployment (1.48) and long-standing health conditions (0.79) stand out as the top predictors for psychological wellbeing. Looking at the same model, the coefficient for group-based arts participation accounts for about 25% of the unemployment effect (0.37/1.48) and 47% of the effect of the long-standing health condition effect (0.37/0.79). While group-based arts participation has a smaller effect than unemployment, it remains substantial. Table IV shows no statistically significant association between solitary arts participation and psychological wellbeing. The sign of the coefficients in frequency of solitary arts participation suggests a potential positive influence on mental health for those participating in arts in solitude once a week or more, compared with less frequent participation. However, no systematic differences were found. Robustness checks in Tables III and IV also confirm the main findings: Life satisfaction and mental health are positively associated with frequent group-based arts participation, but not with solitary participation.

## Discussion

We use UKHLS panel data to investigate whether group-based and solitary arts participation have positive associations with psychological wellbeing. The results show that frequent group-based arts participation is positively associated with mental health, while solitary arts participation shows no such association, regardless of participation frequency. These findings hold after controlling for key social determinants like unemployment and social support.

Our study has two key implications for research on arts participation and mental health. First, our findings show that mental health benefits of arts participation are contingent on its social context. Group-based arts participation provides greater social focus than solitary arts participation, providing opportunities for social bonding and mutual coping. Although the study does not test the effect of specific social mechanisms, the results remain robust across multiple mental health indicators. The results, therefore, partly support previous studies suggesting that art has an independent positive role in mental health [6, 22–24], but also indicate that distinguishing between group-based and solitary participation is a promising direction for the future. Highlighting social context, in conjunction with broader health determinants, provides valuable insights. We found no evidence that solitary arts participation improves outcomes such as life satisfaction; however, unlike earlier research [25], we also found it does not worsen life satisfaction. Second, the study provides insight into effect sizes that are rarely examined in the literature (with exception, Mak et al. [24]). This is a key tool for assessing art’s role in promoting mental health. One estimate [1] suggests that art and cultural activities are the second most important determinant of psychological wellbeing, after diseases. However, viewing art as a singular entity likely overestimates solitary and underestimates group-based arts participation. While we found no large effect size for group-based arts participation, it remains substantial – accounting for 25% of the unemployment effect and 47% of the effect of disabilities/chronic health problems on psychological wellbeing.

The results also have practice implications for designing arts-based social prescriptions [4, 5, 26]. A recent psychological study suggests that being a member of an art group can promote a positive sense of self and social unity [27]. Our study provides systematic evidence of the benefits of group-based arts participation, which may be recommended for those at risk of social isolation such as the elderly. It is possible that even undertaking solitary arts in group settings may increase their beneficial effects. However, caution is needed with group settings, as undesirable dynamics like competition, public criticism and conflict can arise leading to stress and negative emotions [4, 28]. To sustain arts groups, a balanced approach to consider participants’ social needs is recommended.

### Strengths and limitations of this study

The main strength of the research is its use of large-scale longitudinal data, allowing us to study a relatively small segment of the population engaging in group-based or solitary arts participation. This is crucial for art and mental health, given the challenge of synthesising diverse evidence emerging from interviews, clinical trials, and surveys [29]. While qualitative studies provide insights into participant perspectives, large-scale longitudinal data provide systematic evidence to inform public health policies, particularly as arts-based social prescriptions become mainstream in mental health treatment.

There are two main limitations. While our findings emphasise the mental health benefits of group-based arts activities, we cannot determine how results might change if typically solitary activities – such as crafting or painting – were undertaken in socially engaging environments such as clubs, open studios, or broader friendship networks. Additionally, the study cannot identify reverse causality; that is, the effect of mental health on arts participation. For instance, individuals motivated by physical activity may gravitate towards group-based activities like dancing and circus [8, 30], while those with social anxiety may prefer solitary activities.

## Conclusions

Based on longitudinal large-scale data, the results show the mental health benefits of arts participation depend on whether they are done in group-based or solitarily settings. In addition to the intrinsic qualities of arts activities, such as sense of purpose and self-expression, the study provides systematic evidence for practice and further research about the importance of differentiating between group-based and solitary arts participation.

## Supplemental Material

sj-docx-1-sjp-10.1177_14034948251370234 – Supplemental material for The association of solitary versus group-based arts participation with mental health: evidence from the UK Household Longitudinal Study Supplemental material, sj-docx-1-sjp-10.1177_14034948251370234 for The association of solitary versus group-based arts participation with mental health: evidence from the UK Household Longitudinal Study by Gökhan Kaya and Christopher Mathieu in Scandinavian Journal of Public Health
