# Supplementary material for: The association of solitary versus group-based arts participation with mental health: evidence from the UK Household Longitudinal Study
Source: Scand J Public Health. 2025 Oct 3;54(7):777–86. doi: 10.1177/14034948251370234 (PMC13589797; doi:10.1177/14034948251370234)
Supplement: Supplementary file 1 [file sj-docx-1-sjp-10.1177_14034948251370234.docx]

## Supplementary Material

The supplementary material consists of two parts: (1) Robustness Check part II, and (2) the full model specification for Model 2, presented in Table 3 of the manuscript.

### Robustness Check II

This part provides additional analysis based on an alternative measure of solitary arts participation using data from the UK Household Longitudinal Study (UKHLS). All activities included in the UKHLS dataset are listed in Supplementary Table 1.

Our measurement focuses on active participation in the arts—activities in which individuals create or perform as opposed to consuming arts (see Solitary or Group in Supplementary Table 1). Respondents who reported only passive forms of engagement (e.g., reading or attending a book club) do not fit this definition. These cases are labeled as NA in Supplementary Table 1. As noted in the manuscript, some activities, such as playing a musical instrument, do not clearly fall into the solitary-art activities in this study because less than 50% undertake this activity mostly alone [see 15]. While these activities are still more commonly undertaken alone, they often involve significant group participation as well, and can be seen as a hybrid activity. We labelled these (activities 3, 8 and 9) as Solitary* in Supplementary Table 1.

**Supplementary Table 1** *Two sets of variables measuring art activities in the UKHLS (wave 2 and wave 5)*

| Activities-a | Variables | Authors-made classifications |
| --- | --- | --- |
| 1 | Dance, including ballet | Group |
| 2 | Sang to an audience or rehearsed for a performance (not karaoke) | Group |
| 3 | Played a musical instrument | Solitary* |
| 4 | Written music | Solitary |
| 5 | Rehearsed or performed in a play/drama, opera/operetta or musical theatre | Group |
| 5 | Taken part in a carnival or street arts event (e.g. as a musician, dancer or costume maker) | Group |
| 6 | Learned or practised circus skills | Group |
| Activities-b | Variables |  |
| 7 | Painting, drawing, printmaking or sculpture | Solitary |
| 8 | Photography, film or video making as an artistic activity (not family or holidays) | Solitary* |
| 9 | Used a computer to create original artworks or animation | Solitary* |
| 10 | Textile crafts, wood crafts or any other crafts, such as embroidery, knitting, wood turning, furniture making, pottery or jewellery | Solitary |
| 11 | Read for pleasure (not newspapers, magazines or comics) | NA |
| 12 | Written any stories, plays or poetry | Solitary |
| 13 | Been a member of a book club, where people meet up to discuss and share books | NA |

In Supplementary Table 2, we present four alternative measures, including and excluding the items that do not clearly fit within the definition of solitary art activities.

**Supplementary Table 2** Three Alternative Forms of Solitary Arts Participation and a Separate Variable Measuring arts participation in Mixed Settings (Both Solitary and with Others)

| Alternatives | Indicator |
| --- | --- |
| Solitary arts participation (version2) | Solitary arts participation variable+ photography, film + video/digital art (computer based) |
| Solitary arts participation (version3) | Solitary arts participation variable+ play musical instrument |
| Solitary arst participation (version4) | Solitary arts participation variable+ play musical instrument+ photography, film + video/digital art (computer based) |
| Mixed- arts participation  (version 5) | photography, film, or video digital art (animation+ playing musical instrument) |

Solitary arts participation (original measure) refers to the frequency of engaging in art creation through at least one of the following activities: i) writing music; ii) textile crafts, wood crafts, or any other crafts, such as embroidery or knitting; iii) painting, drawing, printmaking, or sculpture; iv) writing stories, plays, or poetry

Supplementary Table 3 includes the dependent variable, psychological wellbeing (GHQ-12 reversed). We enter four different versions of arts participation while keeping the two key control variables, namely attending arts events and the time-effect fixed variable. The aim of these models is to examine whether alternative measures show differences in psychological well-being. We did not further develop the models, as the results did not show statistically significant associations between any of the alternatives and psychological well-being.

**Supplementary Table 3** Fixed effect OLS predicting the association between four different

versions of solitary arts participation / and psychological wellbeing (GHQ-12 [reversed]) without additional controls.

| Solitary arts participation* | Version 2 | Version 3 | Version 4 | Version 5 |
| --- | --- | --- | --- | --- |
| (ref.cat= none) |  |  |  |  |
| once/twice a year | -0.19 | -0.17 | -0.24 | -0.30 |
|  | [-0.50,0.12] | [-0.51,0.18] | [-0.54,0.06] | [-0.68,0.09] |
| at least once a month | -0.20 | -0.18 | -0.20 | -0.31 |
|  | [-0.48,0.09] | [-0.48,0.11] | [-0.48,0.08] | [-0.67,0.05] |
| at least once a week | 0.12 | 0.13 | 0.09 | -0.14 |
|  | [-0.10,0.34] | [-0.10,0.36] | [-0.13,0.31] | [-0.41,0.13] |
| Attending arts events |  |  |  |  |
| (ref.cat= none) |  |  |  |  |
| at least once/twice a year | 0.07 | 0.07 | 0.07 | 0.08 |
|  | [-0.15,0.30] | [-0.15,0.30] | [-0.15,0.30] | [-0.15,0.31] |
| several times a year | 0.22^*^ | 0.22^*^ | 0.22^*^ | 0.23^*^ |
|  | [-0.02,0.47] | [-0.03,0.46] | [-0.02,0.47] | [-0.02,0.48] |
| at least once a month | 0.41^***^ | 0.41^***^ | 0.41^***^ | 0.43^***^ |
|  | 0.07 | [0.11,0.71] | [0.11,0.71] | [0.14,0.73] |
| Wave 5 | -0.10^**^ | -0.10^**^ | -0.10^**^ | -0.11^**^ |
| (ref. cat= Wave 2) | [-0.19,-0.02] | [-0.19,-0.01] | [-0.19,-0.02] | [-0.20,-0.02] |
| Number of observations | 47412 | 47412 | 47412 | 47412 |
| Number of groups | 23706 | 23706 | 23706 | 23706 |
| R-squared for within model | 0.00 | 0.00 | 0.00 | 0.00 |
| ρ | 0.58 | 0.58 | 0.58 | 0.58 |

Note. The results are weighted to correct non-response, and unequal sample selection. The longitudinal weights are provided by UKHLS.

^*^p < 0.05. ^**^ p < 0.01. ^***^ p < 0.001.

* Each column represents different versions of Solitary art participation while utilizing the same dependent variable (GHQ-12 [reversed]). See Supplementary Table 2 for explanations of each version.

### The Full Model Specification (Model 2, Table 3)

Supplementary Table 4 presents all variables used in Model 2 of Table 3 in the manuscript. The table was constructed by sequentially adding one set of control variables in each step. We control frequency of attending art events since those who participate in art activities may engage in art in general and may also attend art events. Although beyond the scope of the current research, the results also show that frequent attendance at art events is consistently associated with greater psychological wellbeing. Presenting full models primarily allows for a clearer understanding of how each set of controls—such as sociodemographic characteristics and social determinants of health—contributes to the model parameters and modifies the association between arts participation and psychological wellbeing. In each model, Supplementary Table 4 shows a positive association between group-based arts participation and psychological well-being.

**Supplementary Table 4** Fixed-effect OLS predicting the association between group-based arts participation and psychological well-being (GHQ-12 (reversed)) with controls (coefficients, confidence intervals in parentheses)

|  | M1 | | M2 | | M3 | | M4 | | M5 | | | M6 | |
| --- | --- | --- | --- | --- | --- | --- | --- | --- | --- | --- | --- | --- | --- |
| Arts participation |  | |  | |  | |  | |  | | |  | |
| (group-based) |  | |  | |  | |  | |  | | |  | |
| (ref.cat= none) |  | |  | |  | |  | |  | | |  | |
| once/twice a year | -0.23 | | -0.24 | | -0.24 | | -0.25 | | -0.27 | | | -0.28 | |
|  | [-0.64,0.18] | | [-0.65,0.17] | | [-0.65,0.17] | | [-0.66,0.16] | | [-0.68,0.14] | | | [-0.69,0.12] | |
| at least once a month | 0.33 | | 0.32 | | 0.32 | | 0.31 | | 0.31 | | | 0.30 | |
|  | [-0.09,0.76] | | [-0.10,0.74] | | [-0.10,0.75] | | [-0.11,0.73] | | [-0.11,0.73] | | | [-0.12,0.72] | |
| at least once a week | 0.43*** | | 0.41*** | | 0.41*** | | 0.41*** | | 0.39*** | | | 0.37*** | |
|  | [0.17,0.68] | | [0.15,0.66] | | [0.16,0.67] | | [0.15,0.66] | | [0.14,0.64] | | | [0.11,0.62] | |
| Wave 5 | -0.09** | | -1.28** | | -1.24** | | -1.21** | | -1.14** | | | -1.11** | |
| (ref. cat= Wave 2) | [-0.18,-0.01] | | [-2.33,-0.24] | | [-2.28,-0.21] | | [-2.24,-0.17] | | [-2.17,-0.11] | | | [-2.14,-0.08] | |
| Attending arts events |  | |  | |  | |  | |  | | |  | |
| (ref.cat= none) |  | |  | |  | |  | |  | | |  | |
| at least once/twice a year | 0.07 | | 0.09 | | 0.07 | | 0.07 | | 0.08 | | | 0.08 | |
|  | [-0.16,0.29] | | [-0.14,0.31] | | [-0.15,0.30] | | [-0.16,0.29] | | [-0.14,0.30] | | | [-0.14,0.30] | |
| several times a year | 0.21* | | 0.22* | | 0.20 | | 0.20 | | 0.19 | | | 0.17 | |
|  | [-0.04,0.45] | | [-0.02,0.46] | | [-0.05,0.44] | | [-0.04,0.44] | | [-0.05,0.42] | | | [-0.06,0.41] | |
| at least once a month | 0.40*** | | 0.39** | | 0.37** | | 0.36** | | 0.33** | | | 0.31** | |
|  | [0.10,0.69] | | [0.09,0.69] | | [0.07,0.66] | | [0.07,0.66] | | [0.04,0.62] | | | [0.02,0.60] | |
| Age |  | | 0.19 | | 0.13 | | 0.12 | | 0.09 | | | 0.09 | |
|  |  | | [-0.17,0.54] | | [-0.22,0.48] | | [-0.23,0.47] | | [-0.26,0.44] | | | [-0.26,0.44] | |
| Age squared |  | | 0.00*** | | 0.00*** | | 0.00*** | | 0.00*** | | | 0.00*** | |
|  |  | | [0.00,0.00] | | [0.00,0.00] | | [0.00,0.00] | | [0.00,0.00] | | | [0.00,0.00] | |
| marital status |  | |  | |  | |  | |  | | |  | |
| (ref. cat= married) |  | |  | |  | |  | |  | | |  | |
| Single |  | | -0.09 | | -0.11 | | -0.11 | | -0.19 | | | -0.21 | |
|  |  | | [-0.61,0.43] | | [-0.63,0.41] | | [-0.63,0.41] | | [-0.70,0.33] | | | [-0.72,0.31] | |
| Divorced |  | | -0.52 | | -0.46 | | -0.43 | | -0.57 | | | -0.58* | |
|  |  | | [-1.21,0.17] | | [-1.15,0.23] | | [-1.12,0.26] | | [-1.25,0.12] | | | [-1.26,0.10] | |
| Widowed |  | | -0.61 | | -0.57 | | -0.52 | | -0.74 | | | -0.76 | |
|  |  | | [-1.57,0.36] | | [-1.54,0.40] | | [-1.48,0.44] | | [-1.72,0.23] | | | [-1.73,0.22] | |
| Child under 16 |  | | 0.24** | | 0.24* | | 0.24* | | 0.18 | | | 0.17 | |
| (ref.cat= no) |  | | [0.00,0+D36.49] | | [-0.01,0.48] | | [-0.00,0.48] | | [-0.06,0.42] | | | [-0.06,0.41] | |
| Employment status |  | |  | | 0.00 | | 0.00 | | 0.00 | | | 0.00 | |
| (ref.cat= employed) |  | |  | | [0.00,0.00] | | [0.00,0.00] | | [0.00,0.00] | | | [0.00,0.00] | |
| unemployed |  | |  | | -1.49*** | | -1.45*** | | -1.47*** | | | -1.48*** | |
|  |  | |  | | [-1.99,-0.99] | | [-1.95,-0.96] | | [-1.96,-0.97] | | | [-1.98,-0.99] | |
| Inactive |  | |  | | -0.34** | | -0.32** | | -0.32** | | | -0.34** | |
|  |  | |  | | [-0.62,-0.05] | | [-0.61,-0.03] | | [-0.61,-0.04] | | | [-0.62,-0.05] | |
| Level of education |  | |  | |  | |  | |  | | |  | |
| (ref. cat: no qualification) |  | |  | |  | |  | |  | | |  | |
| University degree |  | |  | | 0.27 | | 0.26 | | 0.26 | | | 0.27 | |
|  |  | |  | | [-1.52,2.07] | | [-1.54,2.05] | | [-1.50,2.02] | | | [-1.49,2.02] | |
| Other higher degree |  | |  | | 0.66 | | 0.70 | | 0.73 | | | 0.74 | |
|  |  | |  | | [-1.20,2.51] | | [-1.14,2.54] | | [-1.09,2.54] | | | [-1.07,2.55] | |
| A-level etc. |  | |  | | 0.19 | | 0.19 | | 0.20 | | | 0.20 | |
|  |  | |  | | [-1.49,1.87] | | [-1.49,1.87] | | [-1.45,1.85] | | | [-1.44,1.85] | |
| GCSE |  | |  | | 0.15 | | 0.16 | | 0.25 | | | 0.25 | |
|  |  | |  | | [-1.54,1.84] | | [-1.52,1.85] | | [-1.42,1.92] | | | [-1.41,1.91] | |
| Other qualifications |  | |  | | 0.11 | | 0.12 | | 0.29 | | | 0.30 | |
|  |  | |  | | [-1.36,1.58] | | [-1.33,1.58] | | [-1.14,1.72] | | | [-1.14,1.73] | |
| Logged income |  | |  | | 0.14* | | 0.14* | | 0.16* | | | 0.16** | |
|  |  | |  | | [-0.02,0.30] | | [-0.02,0.30] | | [-0.00,0.31] | | | [0.00,0.31] | |
| Longstanding health conditions |  | |  | |  | |  | |  | | |  | |
| Yes (ref.cat=no) |  | |  | |  | | -0.80*** | | -0.80*** | | | -0.79*** | |
|  |  | |  | |  | | [-1.00,-0.60] | | [-1.00,-0.60] | | | [-1.00,-0.59] | |
| Family support | |  | |  | |  | |  | | 0.61*** | 0.61*** | |  |
|  | |  | |  | |  | |  | | [0.48,0.74] | [0.47,0.74] | |  |
| Friend support | |  | |  | |  | |  | | 0.35*** | 0.34*** | |  |
|  | |  | |  | |  | |  | | [0.21,0.49] | [0.20,0.48] | |  |
| Sport activities | |  | |  | |  | |  | |  | 0.06*** | |  |
|  | |  | |  | |  | |  | |  | [0.02,0.10] | |  |
| Number of observations | | 47412 | | 47412 | | 47412 | | 47412 | | 47412 | 47412 | |  |
| Number of groups | | 23706 | | 23706 | | 23706 | | 23706 | | 23706 | 23706 | |  |
| R-squared for within model | | 0.00 | | 0.00 | | 0.01 | | 0.01 | | 0.02 | 0.03 | |  |
| Intraclass correlation (rho) | | 0.58 | | 0.80 | | 0.79 | | 0.77 | | 0.77 | 0.76 | |  |

Note. The results are weighted to correct non-response, and unequal sample selection. The longitudinal weights are provided by UKHLS.

*p < 0.05. ** p < 0.01. *** p < 0.001.
